# The Epidemiological and Economic Impact of a Cell-Based Quadrivalent Influenza Vaccine in Adults in the US: A Dynamic Modeling Approach

**DOI:** 10.3390/vaccines9101095

**Published:** 2021-09-28

**Authors:** Van Hung Nguyen, Yvonne Hilsky, Joaquin Mould-Quevedo

**Affiliations:** 1 VHN Consulting Inc., 95 McCulloch, Montreal, QC H2V 3L8, Canada; vhnguyen@vhnconsulting.com; 2 Seqirus USA Inc., 25 Deforest Avenue, Summit, NJ 07901, USA; yvonne.hilsky@seqirus.com

**Keywords:** influenza 2, cost-effectiveness 3, quadrivalent influenza vaccine 4, cell-based influenza vaccine 5, United States

## Abstract

Mutations of the H3N2 vaccine strain during the egg-based vaccine manufacturing process partly explain the suboptimal effectiveness of traditional seasonal influenza vaccines. Cell-based influenza vaccines improve antigenic match and vaccine effectiveness by avoiding such egg-adaptation. This study evaluated the public health and economic impact of a cell-based quadrivalent influenza vaccine (QIVc) in adults (18–64 years) compared to the standard egg-based quadrivalent influenza vaccine (QIVe) in the US. The impact of QIVc over QIVe in public health and cost outcomes was estimated using a dynamic age-structured SEIR transmission model, which accounted for four circulating influenza strains [A/H1N1pdm9, A/H3N2, B(Victoria), and B(Yamagata)] and was calibrated on the 2013–2018 influenza seasons. The robustness of the results was assessed in univariate and probabilistic sensitivity analyses. Switching from QIVe to QIVc in 18- to 64-year-olds may prevent 5.7 million symptomatic cases, 1.8 million outpatient visits, 50,000 hospitalizations, and 5453 deaths annually. The switch could save 128,000 Quality-Adjusted Life Years (QALYs) and US $ 845 M in direct costs, resulting in cost-savings in a three-year time horizon analysis. Probabilistic sensitivity analyses confirmed the robustness of the cost-saving result. The analysis shows that QIVc is expected to prevent hospitalizations and deaths, and result in substantial savings in healthcare costs.

## 1. Introduction

With an estimated average of 410,000 deaths each year [1], influenza infection is a substantial burden worldwide, despite years of improvement in global immunization policies. In the US, the Centers for Disease Control (CDC) estimates that influenza infections caused illness in 35.5 M people, 16.5 M outpatient visits, 490,600 hospitalizations, and 34,200 deaths during the 2018–2019 winter season alone [2]. Vaccination against influenza is considered the most effective way to prevent influenza infection and its consequences. However, influenza viruses have evolved mechanisms to evade human immune response with antigenic drift and shift phenomena and also develop a diversity of strains that compete for dominant circulation. The complexities seen with influenza viruses are a serious challenge to the effectiveness of immunization campaigns. In recent years influenza vaccination has advanced, with the adoption by some countries (primarily those with more developed economies) of quadrivalent influenza vaccines, which contain antigens for two B lineages rather than just one, and relatively new adjuvanted and high-dose formulations. However, conventional egg-based influenza vaccines may offer suboptimal protection during influenza seasons dominated by influenza A(H3N2) circulation. A recent meta-analysis measured 35% vaccine effectiveness (VE) against A(H3N2) versus 54% to 73% for other strains in working-age adults vaccinated with egg-based influenza vaccines [3]. This poor protection may be partially explained by issues related to the production of egg-based vaccines. Mutations of influenza virus strains during the isolation and propagation steps of egg-based vaccine production may lead to an antigenic mismatch between the influenza candidate vaccine and the circulating strain [4,5]. This issue might be solved by propagating vaccine viruses in mammalian cell lines rather than fertilized chicken eggs. Mammalian cell lines are not subject to egg adaptation, cell-based vaccines have greater antigenic similarity between the original candidate virus and the vaccine virus. In years with a good match between the vaccine and circulating influenza virus strains, the lack of egg adaptation may translate into an increased VE for new cell-based quadrivalent influenza vaccines (QIVc) [6,7].

The assessment of the public health and economic impact of new influenza immunization policies is performed routinely for most countries. Despite WHO recommendations for the assessment of vaccination strategies [8], most of these studies are performed using a simple static epidemiological model [9], which is unable to grasp the indirect effects of vaccination (herd effects). While the potential impact of improved influenza vaccine effectiveness is generally acknowledged for the pediatric population [10], its impact on the adult population remains unclear. The purpose of the present analysis is to evaluate the cost-effectiveness of QIVc in adults (18 to 64 years) compared to conventional egg-based quadrivalent influenza vaccines (QIVe) using a dynamic influenza transmission model able to account for the indirect effect of vaccination.

## 2. Materials

### 2.1. Epidemiology and Vaccine Effectiveness

Influenza incidence estimates were extracted from CDC reports for 5 influenza seasons from 2013 to 2018 (Table S1) [11] and combined with WHO FluNet [12] virological data to obtain yearly incidence per strain for the US. The model assumed 66% of people infected with the influenza virus were symptomatic [13], the virus had an incubation period of 0.8 days, and remained infectious for 1.8 days [14,15].

We used strain-specific QIVe *VE* estimates obtained by Rolfes et al. for the influenza season 2017–2018 [16] assuming *VE* against A(H3N2) was a mismatch between the vaccine and the circulating strain. To estimate *VE* conferred by QIVc, we extracted results from a recent study estimating the relative *VE* (*rVE*) of QIVc compared to QIVe from electronic medical records where patients were matched by propensity score for the influenza season 2017–2018 [17], (i.e., the same influenza season as in Rolfes et al.) [16]. This study estimated an overall *rVE* of 19.3% (95% CI [9.5%;28.0%]) for QIVc compared to QIVe, and age-adjusted *rVE* for the adult population (Table 1). In order to compute the specific QIVc increased effectiveness against A(H3N2) (in 2017–2018, only A(H3N2) antigens are cell-based), we used the total *rVE* estimated on a US cohort comparing QIVc and QIVe for the same influenza season, and then, we recomputed, the QIVc *VE* against A(H3N2) using the following equations:

$$VE_{e}=\;p_{H1N1}*VE_{H1N1}+p_{H3N2}*VE_{H3N2}+p_{BVict}*VE_{BVict}+p_{BYam}*VE_{BYam}\;VE_{c}=\;rVE*\left(1-VE_{e}\right)+VE_{e}\;VE_{c}^{H3N2}=\;\frac{\left(VE_{c}-p_{H1N1}*VE_{H1N1}-p_{BVict}*VE_{BVict}-p_{BYam}*VE_{BYam}\right)}{p_{H3N2}}$$

where *VE_e_* and *VE_c_* stand for the vaccine effectiveness against all influenza viruses of QIVe and QIVc respectively, *p_i_* and *VE_i_* are the proportion of circulating *i* strain and the associated vaccine effectiveness in the influenza season 2017–2018, and $VE_{c}^{H3N2}$ is the specific vaccine effectiveness of QIVc against A(H3N2). We assumed that the QIVc was well-matched against circulating A(H3N2) and not superior to QIVe against A(H1N1) and B strains. Details of QIV_c_ and QIV_e_ *VE* per age group from studies conducted during the 2017–2018 influenza season are given in Table 1 [16,17].

### 2.2. Epidemiological and Economic Model

**Figure 1:**
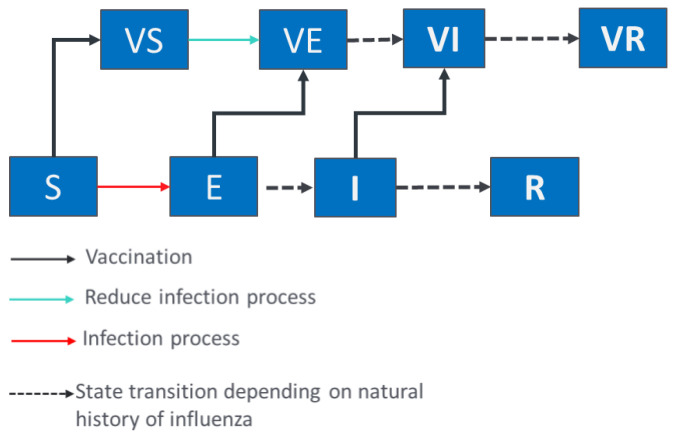
Diagram of the influenza transmission model. S_i_, E_i_, I_i_, and R_i_ stand for susceptible, exposed, infectious, and recovered individuals respectively regarding the influenza strain *I*; VS_i_, VE_i_, and VI_i_ stand for susceptible, exposed and infectious individuals experiencing a non-protective vaccination respectively regarding influenza strain *i*, VR_i_ are individuals protected and vaccinated against influenza strain; *i* stands for influenza strains: A/H1N1, A/H3N2, B Victoria, B Yamagata.

A four strains compartmental transmission model was developed to provide estimates of the epidemiological impact of the switch from QIVe to QIVc. The model is a classic SEIR model where the population can be either Susceptible to infection with Influenza strain *I* (*S_i_*), Exposed to the strain (*E_i_*), Infected and infectious (*I_i_*), or Recovered from infection (*R_i_*) (Figure 1). The vaccinated population could still be infected and contribute to the infection dynamic but with a reduced probability corresponding to the vaccine effectiveness against the given influenza strain. The model simulates independently the epidemiological dynamics of A(H1N1)pdm09, A(H3N2), B/Victoria, and B/Yamagata for a given influenza season. The model is structured by age-group (6 to 23 months, 2 to 4 years, 5 to 12 years, 13 to 17 years, 18 to 49 years, 50 to 64 years, and more than 65 years) and uses a contact matrix to account for the assortative rate of contacts between age-groups. In our analysis, we used in our base case analysis the matrix from Mossong et al. [18] and have conducted a sensitivity analysis using Zagenhi et al. [19]. Both matrices have provided qualitatively similar results. Probabilities of influenza transmission per influenza strain are estimated for each influenza season to match the US reported strain specific attack rate (2013 to 2018 influenza seasons). We assume a pre-immunity of a third of the population based on estimations from Baguelin et al. [20]. The estimation process uses the non-linear Nelder-Mead simplex algorithm [21] to maximize a likelihood function.

The economic model is based on a decision tree model published in De Boer et al. [22] whose inputs are given in Table S2. We used the same methodology and the same health outcomes computed on the whole population. The number of cases per age-group estimated by the epidemiological model, shown in Table 2, are taken as inputs of the economic model. Probabilities of general practitioner visit, hospitalization, and death are applied to the number of cases attributed to a high- or low-risk group and then translated into public health outcomes and costs. The economic analysis is performed from a societal perspective without taking into account productivity loss due to death. We consider a willingness to pay per QALY threshold of US$50,000 to consider a strategy as cost-effective [23].

### 2.3. Economic Data

Disease costs and QALYs were extracted from a recent influenza health economic analysis performed in the US context [22]. The cost of a workday for the pediatric population is assumed to be related to parental work loss. Vaccine costs for QIVe and QIVc were set at $17.22 and $24.22, respectively [24]. We do not consider administration costs since they are assumed to be the same across the different vaccination strategies (no difference in vaccination coverage). Details of the costs per age-groups are given in Table S2.

### 2.4. Scenarios

As a reference strategy, we assume that the US population is vaccinated with conventional QIVe for those aged under 65 years of age and TIV HD for those aged 65 years and above. Then we compare this strategy to a scenario where QIVe is replaced by QIVc for people aged 18 to 64 years, other age-groups keeping their baseline vaccination. For both scenarios, we use age-based vaccination coverage rate documented by CDC [25], in particular, we consider that 34.9% of people aged 18 to 49 years, 47.30% of people aged 50 to 64 years, and 68.10% of people older than 65 years are vaccinated against influenza (Table S3). In this base case comparison, we consider that a seasonal mismatch between the QIVe A(H3N2) strain and the circulating strain due to egg adaptation occurred during the last 3 years out of 5 years in the analysis scope [26]. As a sensitivity analysis, we also assessed the impact of QIVc when the mismatch due to egg adaptation occurred over a varying number of years from 1 to 5 years and randomly picking the influenza season with a mismatch.

We also performed a stochastic probabilistic sensitivity analysis in order to assess the robustness of our results regarding uncertainties in vaccine effectiveness, economic inputs (primary care and hospitalization costs), probability of outcomes, listed in Tables S2 and S4, with their probability distributions. In this analysis, 1000 sets of the above-mentioned parameters are randomly drawn from distributions indicated in Tables S2 and S3. Clinical and economic results are averaged over the 5 influenza seasons.

## 3. Results

In our base case scenario of three seasonal A(H3N2) mismatches due to egg adaptation, our analysis shows that using QIVc instead of QIVe in the 18- to 64-year-old population would have prevented 5.7 M cases of influenza, 1.8 M GP visits, almost 50 K hospitalizations, and more than 5400 deaths. In total, QIVc would have saved US $ 845 M in direct costs and saved 128 K QALYs. Hence, the switch from QIVe to QIVc in the adult population would be a cost-saving strategy (Table 2).

**Figure 2:**
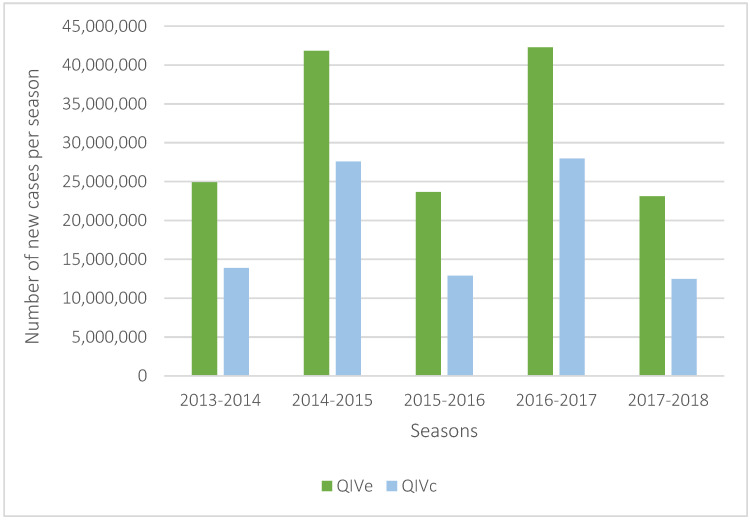
Number of influenza cases in the overall US population over the 5 studied seasons considering systematic A(H3N2) mismatch.

**Figure 3:**
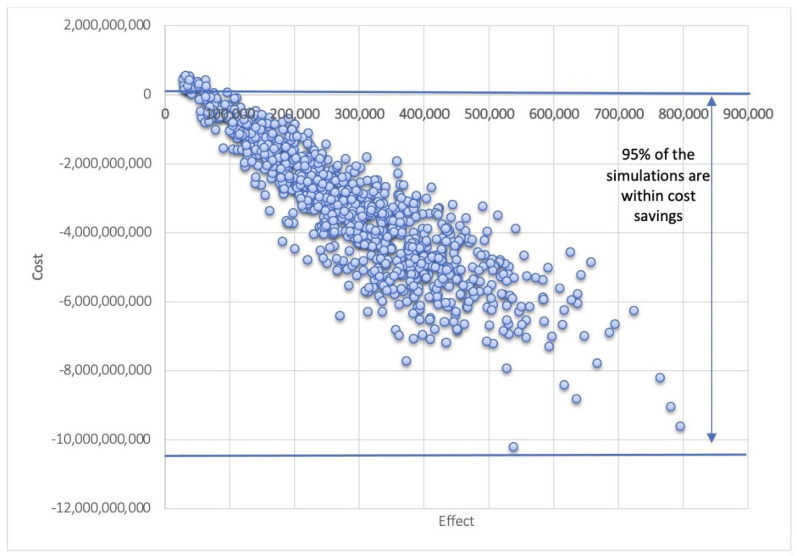
Probabilistic sensitivity analysis of the QIVc scenario compared to QIVe. Health is measured in QALY and cost in US$.

Over five influenza seasons of A(H3N2) mismatch, using QIVc instead of QIVe in the 18 to 64 years population would have a substantial effect but irregular effect, depending on the influenza season, depending on A(H3N2) seasonal circulation (Figure 2). Hence, the choice of the influenza season was also randomly varied in the probabilistic analysis to assess the uncertainty related to the epidemiological context. Varying the number of influenza seasons with an A(H3N2) mismatch of egg-based vaccines between one and five years still show that QIVc would be cost-saving or very cost-effective (Table 3). Probabilistic sensitivity analysis confirms that 95% of the 1000 simulations gave a cost-saving result (Figure 3).

## 4. Discussion

This analysis emphasizes the potentially major public health gain, which could be achieved using QIVc. Several studies have highlighted the suboptimal VE of egg-based vaccines against some strains of A(H3N2) [6,27]. While the link between VE reduction and egg-related strain mutations is still poorly understood [28] and remains to be fully investigated, recent studies in different populations tend to confirm the clinical benefits [7] of QIVc over QIVe regarding A(H3N2) vaccine strain mismatch. Assessing the strain-specific rVE of QIVc compared to QIVe remains a difficult challenge as egg-adaptation phenomena, while most common on A(H3N2), may also occur on B lineages. However, we used QIVc rVE estimated during the 2017–2018 influenza season, when only the A(H3N2) component of QIVc had been grown in cells, and we derived our strain specific VE estimations only from this influenza season. Our analysis relies on retrospective studies [7,17] performed during the 2017–2018 influenza season when A(H3N2) represented 66% of the influenza positive samples [12]. By construction, these analyses were only able to assess non-strain specific QIVc rVE compared to QIVe, and we had to estimate QIVc strain specific VE, assuming that the increased total VE was only linked to an increase of VE against A(H3N2). Also, variations in QIVc rVE are likely to occur due to the changing distribution patterns of circulating influenza strains. Namely, influenza seasons with a highly dominant A(H1N1)pdm09 circulation (2015–2016) will see a low benefit to QIVc compared to QIVe, while others, like 2014–2015, 2016–2017, or 2017–2018, may see a significant one. Hence, analysis on multiple influenza seasons is necessary, in order to fully assess the potential “averaged” impact of cell-based vaccines across various realistic epidemiological contexts.

We chose as a base case scenario that a mismatch occurred between the A(H3N2) circulating strain and the egg-based vaccine strain during three influenza seasons. We considered this choice a median scenario between a systematic yearly mismatch and no mismatch at all. In addition, this assumption has a limited impact on our results since (1) we consider the observed influenza strain distribution, and (2) we have analyzed situations when the number of mismatched years was varied from one to five years and reached qualitatively similar results. In addition, our results are consistent with previous health-economic analyses of QIVc in Europe (UK, Spain, Italy, Germany) [29,30,31], where QIVc has been shown to be either cost-saving or highly cost-effective.

Our analysis uses a four-strain SEIR compartmental model with an age-structure. This kind of approach, previously used in several similar analyses [9,32,33,34], is key to capturing the potential indirect effects of influenza vaccination, accounting for prevented chains of transmission. However, it relies on assumptions about age-related contact rates, which may be difficult to measure for specific countries. Despite its advantages, our approach suffers from limitations inherent to any modeling exercise. In absence of better estimates, our transmission model assumes that 30% of the population benefits from remaining immunity against influenza based on a British modeling study [14]. In addition, uncertainties regarding surveillance-based influenza incidence estimates, or influenza strains circulations in the US will directly impact the epidemiological dynamics reproduced by the model.

Finally, our analysis shows the potential public health benefits of the use of QIVc in the 18- to 64-year-old population in the US during the 2013–2018 influenza seasons. Of course, results from retrospective analyses may be different from what will be seen as QIVc vaccines become more common in future years because of the challenge in predicting influenza strain circulation. Nevertheless, given the ongoing burden of the coronavirus 2019 (COVID-19) pandemic on healthcare systems and the economy, the US Advisory Committee on Immunization Practices (ACIP) recommends influenza vaccination as an important tool to reduce stress on those systems. In addition, QIVc is now also recommended for the pediatric population as well as adults, which can be expected to reinforce the positive impact of influenza vaccination [35]. Vaccination of young children not only reduces the potential household transmission but also could reduce the indirect burden of influenza due to missed work by parents [36,37,38,39]. To facilitate uptake, the ACIP recommends the coadministration of influenza and COVID-19 vaccines for all individuals eligible for both vaccines [35].

## 5. Conclusions

This study demonstrates the potential public health benefits of QIVc in the adult population in the US. The use of QIVc could be clinically superior and offer substantial cost-savings compared to the current vaccination standard of QIVe. Sensitivity analyses on VE, costs, and the number of influenza seasons with egg-based strain mismatch show the robustness of QIVc cost-effectiveness.

## Figures and Tables

**Table 1:** Vaccine effectiveness (VE) for QIVe and QIVc, per age-group, based on estimates of relative vaccine effectiveness (rVE) between the two vaccines [16,17].

	Match [16] (CI 95%)			Mismatch [16] (CI 95%)	Match (Computed)	rVE [17]
Age Group (Years)	A/H1N1pdm09	B Victoria	B Yamagata	A(H3N2)	A(H3N2)	
18–49	48% (18–67%)	57% (43–69%)	57% (43–69%)	14% (0–30%)	43%	26.8% (14–37%)
50–64	36% (0–67%)	44% (24–60%)	44% (24–60%)	21% (0–41)	50%	

Note: We report 95% confidence intervals (CI) in parenthesis. We have assumed a 0% in the lower bound 95% CI in vaccine effectiveness when negative percentage was reported by Rolfes et al. [16]. We used VE estimates against influenza B (all lineages), as age-specific estimates were not available by B lineages.

**Table 2:** Base case result—3 years of mismatch out of 5 years. The 3 years of mismatch considered are the 3 most recent influenza seasons.

	QIVe	QIVc		Difference
Number of doses	118,818,800	118,818,800		0
Number of cases	27,240,600	21,556,900		−5,683,700
Number of GP * visits	8,595,300	6,751,400		−1,843,900
Lost workdays	29,390,500	22,983,500		−6,407,000
Hospitalizations	199,916	150,191		−49,725
Deaths	22,436	16,983		−5,453
Life years lost	371,040	277,635		−93,405
Life years lost (discounted)	269,745	202,268		−67,477
QALY lost due to sickness	165,410	129,151		−36,259
QALY lost due to death	367,118	274,906		−92,212
QALY lost due to death (discounted)	266,001	199,575		−66,426
Total QALY lost	532,527	404,057		−128,470
Total QALY lost (discounted)	431,410	328,726		−102,684
Cost of GP visits	1,028,822,400	775,428,700		−253,393,700
Cost of hospitalizations	3,794,189,100	2,775,660,700		−1,018,528,400
Cost of lost workdays	2,105,378,600	1,648,894,900		−456,483,700
Vaccine cost	2,610,747,400	3,037,887,500		427,140,100
Total direct costs	7,433,759,000	6,588,976,900		−844,782,100
ICER			−10,400 [−17,400; 11,000] (cost saving)	

* General practitioner.

**Table 3:** Base case result—3 years of mismatch out of 5 years. The 3 years of mismatch considered are the 3 most recent influenza seasons.

	Medical Costs *			QALY ** Gained			ICER ***		
Number of Mismatch Years	Median	Lower Bound	Upper Bound	Median	Lower Bound	Upper Bound	Median	Lower Bound	Upper Bound
1	−136,179,800	−2,176,061,100	821,374,700	74,800	10,300	205,900	−1800	−12,000	67,900
2	−1,570,682,800	−4,916,394,100	577,624,600	164,100	26,800	396,300	−8600	−16,000	15,000
3	−2,749,808,800	−7,530,202,100	351,732,800	241,200	42,200	600,100	−10,500	−18,000	3400
4	−3,999,641,400	−9,658,482,100	182,466,200	325,700	50,400	732,600	−11,600	−18,800	100
5	−5,222,697,300	−12,426,192,400	21,380,200	412,000	64,600	920,200	−12,200	−19,400	−3200

* Medical costs include cost of GP visits and hospitalizations; ** Quality-adjusted life year; *** Incremental cost-effectiveness ratio.

## Data Availability

The CDC data analyzed in this study are available online at: https://www.cdc.gov/flu/weekly/pastreports.htm, accessed on 17 September 2021.

## Supplementary Materials

The following are available online at https://www.mdpi.com/article/10.3390/vaccines9101095/s1, Table S1: Percentage of strains circulation in the United States from 2013 to 2018 reported by the Centers for Disease Control and Prevention in the Weekly US Influenza reports; Table S2: Influenza economic et clinical outcome parameters; Table S3: Influenza vaccination coverage rate in the US in 2018–2019 according to the Centers for Disease Control and Prevention. Flu Vaccination Coverage, United States, 2018–19 Influenza Season; Table S4: Vaccine effectiveness, relative effectiveness distribution probabilities. Random draws are bounded to 0% and 100%, mean and standard deviation are computed from the bounds of the 95% CI.
