# Supplementary material for: The Epidemiological and Economic Impact of a Cell-Based Quadrivalent Influenza Vaccine in Adults in the US: A Dynamic Modeling Approach
Source: Vaccines (Basel). 2021 Sep 28;9(10):1095. doi: 10.3390/vaccines9101095 (PMC8538069; doi:10.3390/vaccines9101095)
Supplement: Supplementary file 1 [file vaccines-09-01095-s001.zip › vaccines-1371410-supplementary.pdf]

Table S1: Percentage of strains circulation in the United States from 2013 to 2018 reported by the Centers for Disease Control and Prevention in the Weekly US Influenza reports.

Season	A/H1N1	A/H3N2	B Yamagata	B Victoria
**2013-2014**	66.7%	20.5%	6.0%	6.7%
**2014-2015**	0.5%	81.3%	9.1%	9.1%
**2015-2016**	62.0%	18.1%	6.3%	13.6%
**2016-2017**	2.5%	78.6%	5.3%	13.6%
**2017-2018**	11.8%	66.0%	2.4%	19.9%

Table S2: Influenza economic and clinical outcome parameters

Item	Distribution	mean	Param1	Param2
**number of GP visits per case LR in 18-49**	Beta	0.31	338	752
**number of GP visits per case LR in 50-64**	Beta	0.31	338	752
**number of GP visits per case HR in 18-49**	weight	2		
**number of GP visits per case HR in 50-64**	weight	2		
**number of hospitalizations per cases LR in 18-49**	Beta	0.0042	9	2124
**number of hospitalizations per cases LR in 50-64**	Beta	0.0193	8.9	452
**number of hospitalizations per cases HR in 18-49**	Beta	0.0042	9	2124
**number of hospitalizations per cases HR in 50-64**	Beta	0.0193	8.9	452
**number of deaths per cases LR in 18-49**	Beta	0.0001	9	99981
**number of deaths per cases LR in 50-64**	Beta	0.0013	8.9	6599
**number of deaths per cases HR in 18-49**	Beta	0.0001	9	99981
**number of deaths per cases HR in 50-64**	Beta	0.0013	8.9	6599
**GP cost per consultation LR in 18-49**	Lognormal	158	5	0.03
**GP cost per consultation LR in 50-64**	Lognormal	190	5.2	0.04
**GP cost per consultation HR in 18-49**	Lognormal	918	6.8	0.036
**GP cost per consultation HR in 50-64**	Lognormal	928	6.8	0.018
**Hospital cost per episode LR in 18-49**	Lognormal	24076	10	0.22
**Hospital cost per episode LR in 50-64**	Lognormal	28245	10	0.24
**Hospital cost per episode HR in 18-49**	Lognormal	60419	11	0.2
**Hospital cost per episode HR in 50-64**	Lognormal	52300	11	0.075
**QALY loss per nonfatal ILI case in 18-49**	Normal	0.007	0.007	0.0008
**QALY loss per nonfatal ILI case in 50-64**	Normal	0.007	0.007	0.0008
**QALY loss per hospitalization in 50-64**	Normal	0.013	0.013	0.0015
**QALY loss per hospitalization in 65+**	Normal	0.013	0.013	0.0015
**Lost Workday cases in 18-49**	Gamma	0.5	3.8	0.13
**Lost Workday cases in 50-64**	Gamma	0.5	3.8	0.13
**Lost workday outpatient visit LR in 18-49**	Gamma	1	3.8	0.26
**Lost workday outpatient visit LR in 50-64**	Gamma	2	3.8	0.52
**Lost workday outpatient visit HR in 18-49**	Gamma	2	3.8	0.52
**Lost workday outpatient visit HR in 50-64**	Gamma	4	3.8	1
**Lost workday outpatient visit HR in 65+**	Gamma	7	7	1.8
**Lost Workdays Hosp LR in 18-49**	Gamma	12	3.8	3.1
**Lost Workdays Hosp LR in 50-64**	Gamma	13	3.8	3.4
**Lost Workdays Hosp HR in 18-49**	Gamma	21	15.94	1.4
**Lost Workdays Hosp HR in 50-64**	Gamma	24	17.3	1.47

Note: For Beta distribution, param1 and param2 stand for the shape parameters a and b, for Lognormal they stand for the log of the mean and log of the standard deviation, for normal distributions they stand for the bounds of the 95%CI, and for Gamma distributions they stand for the shape and scale parameters.

Table S3: Influenza vaccination coverage rate in the US in 2018-2019 according to the Centers for Disease Control and Prevention. Flu Vaccination Coverage, United States, 2018–19 Influenza Season. Available from: https://www.cdc.gov/flu/fluvaxview/coverage-1819estimates.htm.

Age group	US vaccination coverage rate in 2018-2019
**6-23 m**	73.40%
**2-4yrs**	73.40%
**5-12yrs**	63.60%
**13-17yrs**	52.20%
**18-49yrs**	34.90%
**50-64yrs**	47.30%
**65+ yrs**	68.10%

Table S4: Vaccine effectiveness, relative effectiveness distribution probabilities. Random draws are bounded to 0% and 100%, mean and standard deviation are computed from the bounds of the 95%CI.

	Age	**Item**	**Distribution**	**lower bound of 95%CI**	**upper bound of 95%CI**
**Vaccine effectiveness match**	18-49	A/H1N1pdm09	Normal	18%	67%
		A/H3N2	Normal	0%	93%
		B Victoria	Normal	43%	69%
		B Yamagata	Normal	43%	69%
	50-64	A/H1N1pdm09	Normal	0%	67%
		A/H3N2	Normal	0%	98%
		B Victoria	Normal	24%	60%
		B Yamagata	Normal	24%	60%
**Vaccine effectiveness mismatch**	18-49	A/H3N2	Normal	0%	41%
	50-64	A/H3N2	Normal	0%	39%
**RVE**	18-49	-	Normal	14%	37%
	50-64	-	Normal	14%	37%
